# Mini-open carpal tunnel release: technique, feasibility and clinical outcome compared to the conventional procedure in a long-term follow-up

**DOI:** 10.1038/s41598-022-11649-z

**Published:** 2022-06-01

**Authors:** Angelika M. Schwarz, Georg Lipnik, Gloria M. Hohenberger, Aurel Krauss, Michael Plecko

**Affiliations:** 1 AUVA – Trauma Hospital (UKH) Styria | Graz, Teaching Hospital of the Medical University of Graz, Göstinger Straße 24, 8020 Graz, Austria; 2 Department of Orthopaedics and Trauma, Medical University of Graz, Auenbruggerplatz 5, 8036 Graz, Austria; 3 Division of Macroscopic and Clinical Anatomy, Gottfried Schatz Research Centre, Medical University of Graz, Harrachgasse 21, 8010 Graz, Austria; 4 Department of Trauma Surgery, State Hospital Feldbach – Fürstenfeld, Feldbach, Ottokar-Kernstock Straße 18, 8338 Feldbach, Austria

**Keywords:** Peripheral neuropathies, Orthopaedics, Chronic pain, Neurological manifestations, Autonomic nervous system

## Abstract

We sought to evaluate the findings of our anatomically landmarks based mini-open procedure (MCTR) through a palmar approach and to compare its outcome and practicability to the conventional method (OCTR). The study consisted of 100 matched patients (n = 50 MCTR, n = 50 OCTR) with a minimum follow-up of three years. The outcome was characterized via the Disabilities of Arm, Shoulder and Hand Score (DASH), Symptom Severity Scale (SSS), Functional Status Scale (FSC), and Visual Analogue Scale (VAS). All adverse events were observed. An alpha of 0.05 and a confidence level of 95% were set for statistical analyses. Both techniques showed comparable functional results in a long-term period (mean follow-up MCTR: 60 months and OCTR: 54 months). MCTR versus OCTR at mean: DASH: 4.6/8.3 (p = 0.398), SSS: 1.3/1.2 (p = 0.534), FSC: 1.3/1.2 (p = 0.617), VAS: 0.4/0.7 (p = 0.246). The MCTR convinced through a lower rate of scar sensibility (MCTR: 0% vs. OCTR: 12%, 0/50 vs. 6/50; p = 0.007) and pillar pain, as well as a shortened recovery period and surgical time relative to the OCTR. Low complication rates were observed in both groups, no recurrences had to be documented. The MCTR procedure revealed a similar good clinical outcome as the conventional technique. MCTR is a minimally-invasive, reliable, fast and simple procedure with an obvious benefit regarding scar sensibility.

## Introduction

The carpal tunnel syndrome (CTS) is the most frequently encountered compressive neuropathy^1–6^ with a reported prevalence of 3.8% in the general population^7^. The described prevalence of CTS varies according to the used diagnostic criteria^7^. It is well known that certain risk factors^8^ as well as occupational factors influence^9^ its prevalence.

Following diagnostics via characteristic symptoms and electroneurography, a conservative treatment algorithm including splinting, non-steroidal anti-inflammatory drugs and local corticosteroid injections is usually initiated^3,5,10^. In cases of failure of the conservative regime, surgical carpal tunnel release (CTR) is warranted. Open carpal tunnel release (OCTR) is the commonly accepted method^3^. Although this procedure enables direct visualization, reliable division of the flexor retinaculum and the ability to identify anatomical variations; it includes the possibility of postoperative wound pain, scar sensibility as well as pillar pain^2^. To overcome these complications, several endoscopic and mini-incision approaches have been developed in the recent years^2,11^. Endoscopic carpal tunnel release may be performed as a single- or double-portal technique^5^. It has been reported to evoke reduced postoperative pain^5^, fewer wound-related complications (including scar sensibility, pillar pain or hypertrophic scar formation) and earlier return to work and activities of daily living^5,10,12^. However, it includes an increased risk for median nerve and vascular injury or incomplete division of the flexor retinaculum^2,10^. Mini-open carpal tunnel release (MCTR) combines the advantages of both techniques and has been reported to have low complication rates^13^.

The aim of this study was to evaluate the long-term follow-up of a MCTR based on previously described anatomically landmarks^14^ via a palmar approach, and to compare it to the conventional OCTR. Therefore, we hypothesized that the safety and practicability between MCTR and OCTR is similar. More specifically, we sought to determine if MCTR is superior to traditional techniques in terms of patient satisfaction and functional recovery.

## Material and methods

### Patient collective

Fifty patients, who had undergone unilateral MCTR during the time interval between 1st January 2008 and 31st December 2015 at our Level III trauma center, were included in the study. Fifty patients, who had received conventional OCTR during the same time stage, were matched regarding age and sex to the MCTR group. Each surgical group had been treated by one experienced trauma consultant and hand specialist (MCTR group: MP, OCTR group: AK). The type of operative procedure was not randomized. A minimum follow-up period between surgery and patient visit of three years was defined for final reevaluation.

The diagnosis of CTS was founded on the occurrence of sensory disturbances and/or weakness alongside the supply territory of the median nerve as well as a distinctive anamnesis of pain. All patients obtained a pre-operative electromyogram testifying median nerve neuropathy. Conservative treatment including previous physiotherapy and splint application failed in all patients.

Exclusion criteria involved local or systematic signs of inflammation, distortion of anatomy, neurologic or soft tissue defects, former wrist and hand surgeries as well as bilateral CTS symptomatic and durance of symptoms of more than one year. Patients with other co-morbidities like insulin dependent diabetes mellitus, polyneuropathies, smoking or rheumatoid arthritis were omitted.

### Surgical techniques

A standardized scheme was used for both groups. Surgery was performed in a consistent technique per group, any specific differences per surgical procedure are described below. All surgeries were performed in the operation theatre in regional anesthesia and by using an upper arm tourniquet. All hands were placed in supine position and fixed with a lead hand splint. A mini-suction drainage was inserted and systematically left in place for one day. Wound-closure was performed using non-absorbable 5.0 sutures {Polyamide, ETHILON 668H, Johnson & Johnson Medical GmbH, Norderstedt, Germany} without the use of Leukostrips. All wounds were covered with a Gauze Swab {Lohmann & Rauscher International GmbH & Co. KG, Rengsdorf, Germany} for direct compression. Before releasing the upper arm tourniquet, a dressing was applied consisting of Gauze Swabs as a padding roll following a medium-stretch Compression Bandage {Lenkelast, Lohmann & Rauscher International GmbH & Co. KG, Rengsdorf, Germany}. After the surgical procedure, the hands were kept in this fist bandage for one day. Early dressing removal, including mini-suction drainage removal and clinical control were conducted via the surgeon 24 h postoperatively. Then, a plaster {Cosmopor, Hartmann AG, Heidenheim, Germany} was applied and the patients were discharged. The free-functional aftercare was started on the first postoperative day, avoiding heavy weight lifting for two weeks. The sutures were removed 14 days following surgery (first follow up).

#### OCTR

Concerning the OCTR technique, a longitudinal incision of 3.5 cm was performed alongside the median palmar crease in a proximal direction and stopped 0.5 cm distally to the wrist crease. Following subcutaneous dissection, the transverse carpal ligament, as well as the thenar branch of the median nerve, were depicted and the ligament was cut at its ulnar border in a proximal direction.

#### MCTR

Regarding the MCTR group, both styloid processes were palpated and connected with a horizontal line by use of a pen. Then, a parallel line was drawn in the palm from the most proximal extend of the first web space, named Kaplan’s cardinal line. This line was addressed as a surface marker for a safe zone to the superficial palmar arch in the literature previously^15–17^.

**Figure 1:**
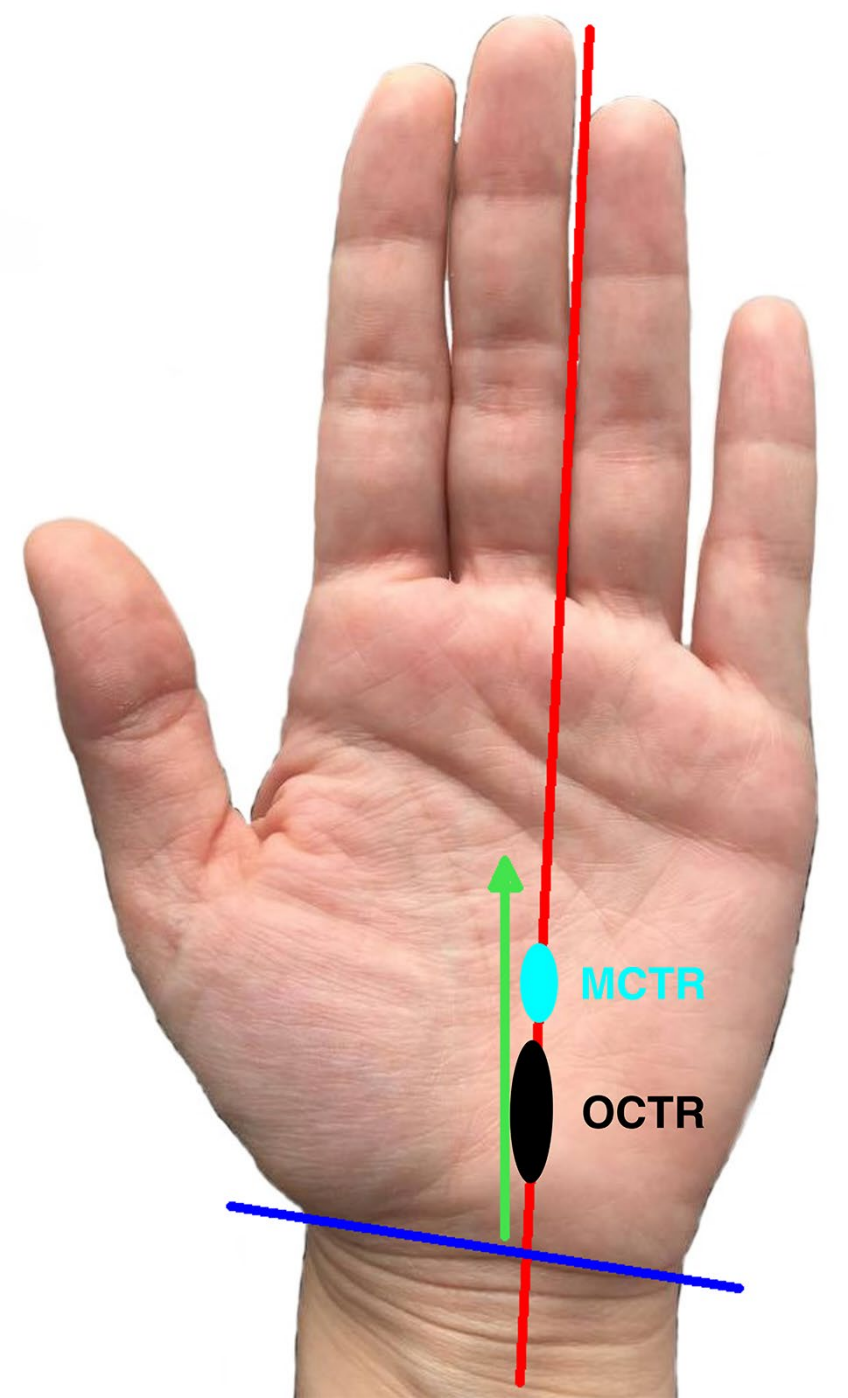
Schematic depiction of MCTR and the skin incisions of both techniques: The blue circle area represents the skin incision of the MCTR, the black circle the skin incision area of the OCTR. The red line represents the longitudinal orientation line to the ring finger, blue marked is the styloid horizontal line. The green line displays the maximal dimension of the transverse carpal ligament as Hohenberger et al. have described^14^. *MCTR* mini-open carpal tunnel release, *OCTR* Open carpal tunnel release.

**Figure 2:**
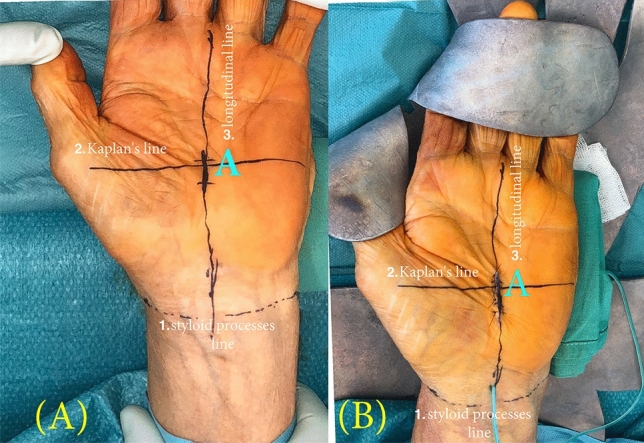
(**A**) Preoperative planning and (**B**) Postoperative result of MCTR: The following lines are drawn: 1. Horizontal line between both styloid processes 2. Horizontal parallel line—the Kaplans cardinal line: 3. Longitudinal line on the ring finger’s radial side. (**A**) The intersection point of these two lines (2. and 3.) marks the reference point for the skin incision. (**B**) The suture closuring of the mini-open procedure—which extends not more than 1–1.5 cm—and its localization are pictured. *MCTR* mini-open carpal tunnel release.

Next, a perpendicular longitudinal line was drawn on the ring finger’s radial side. This line ends directly over the tendon of the palmaris longus muscle on the distal forearm, if existing. The intersection point of these lines in the palm was used as reference point (A). The skin incision extends the one-third from the reference point distally, the other two-thirds in a proximal direction (see Figs. 1 and 2).

**Figure 3:**
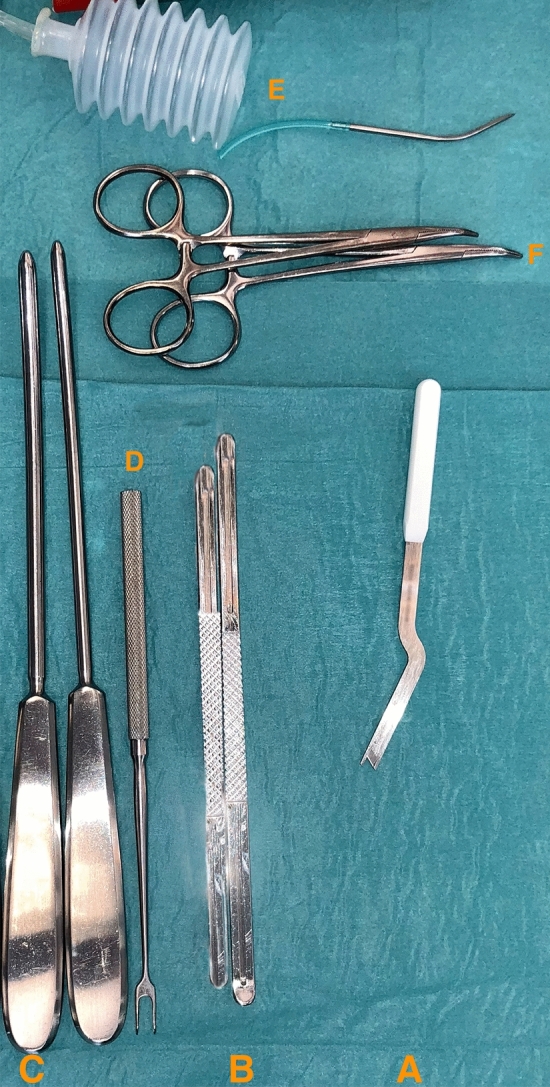
The instrumental system for MCTR: It consists of a two-component system: A. the special cutting knife, B. the protective guide. {SafeGuard Mini Carpal Tunnel Release System, Art.-No. 08-0001 and 08-0003, INTEGRA LifeSciences Corporation, USA}. The “Hegar” dilatators are marked with C. {KARL STORZ SE & Co. KG, Art.-No. 28147 SA, Germany} D. Fomon retractor, E. mini suction drain, F. Mosquito forceps. {MEDICON eG, Art.-No. 20.50.05, Germany; MEDICOPLAST International GmbH, Art.-No. 770 (107867), Germany; MEDICON eG, Art.-No. 15.45.12, Germany}. *MCTR* mini-open carpal tunnel release.

**Figure 4:**
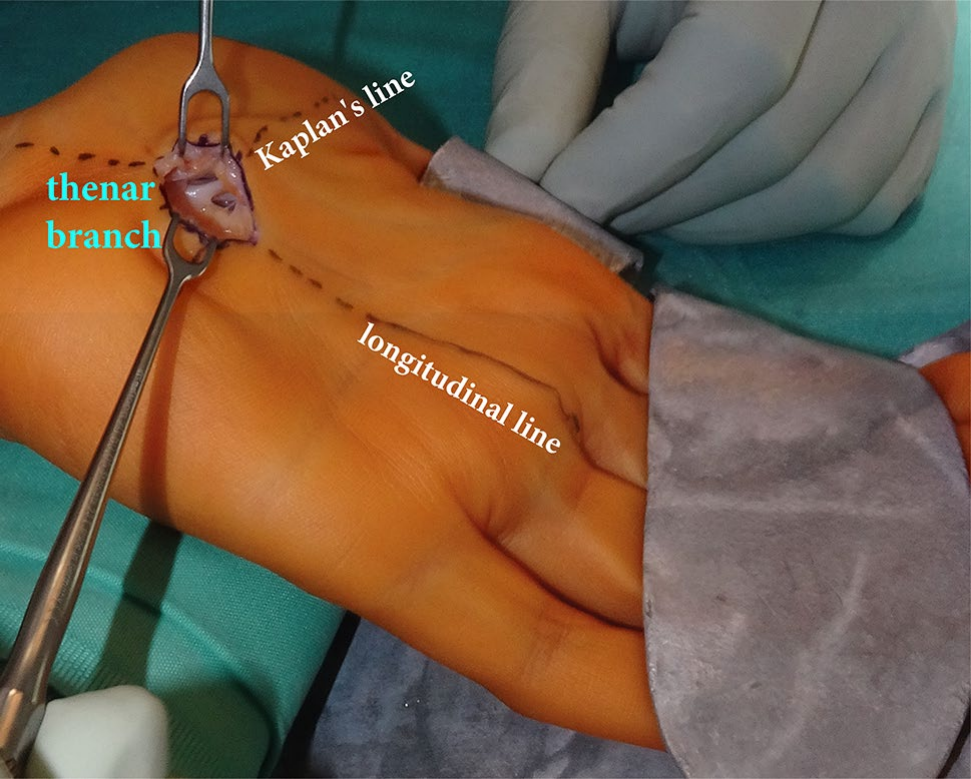
Intraoperative picture of MCTR: The exit point of the thenar branch of the median nerve is fully visible in the surgical field. The superficial palmar artery arch is protected and visible through the skin incision and the proximal direction of the split. {MEDICON eG, Art.-No. 20.50.05, Germany}. *MCTR* mini-open carpal tunnel release.

A 1–1.5 cm palmar incision was performed in a longitudinal fashion proximally, passing in every case the thenar crease from ulnar. Following the opening of the palmar aponeurosis, the transverse carpal ligament was tunneled above and below itself in a proximal direction with Hegar dilatators (see Fig. 3). The superficial palmar arterial arch as well as the thenar branch of the median nerve were depicted and protected (see Fig. 4).

**Figure 5:**
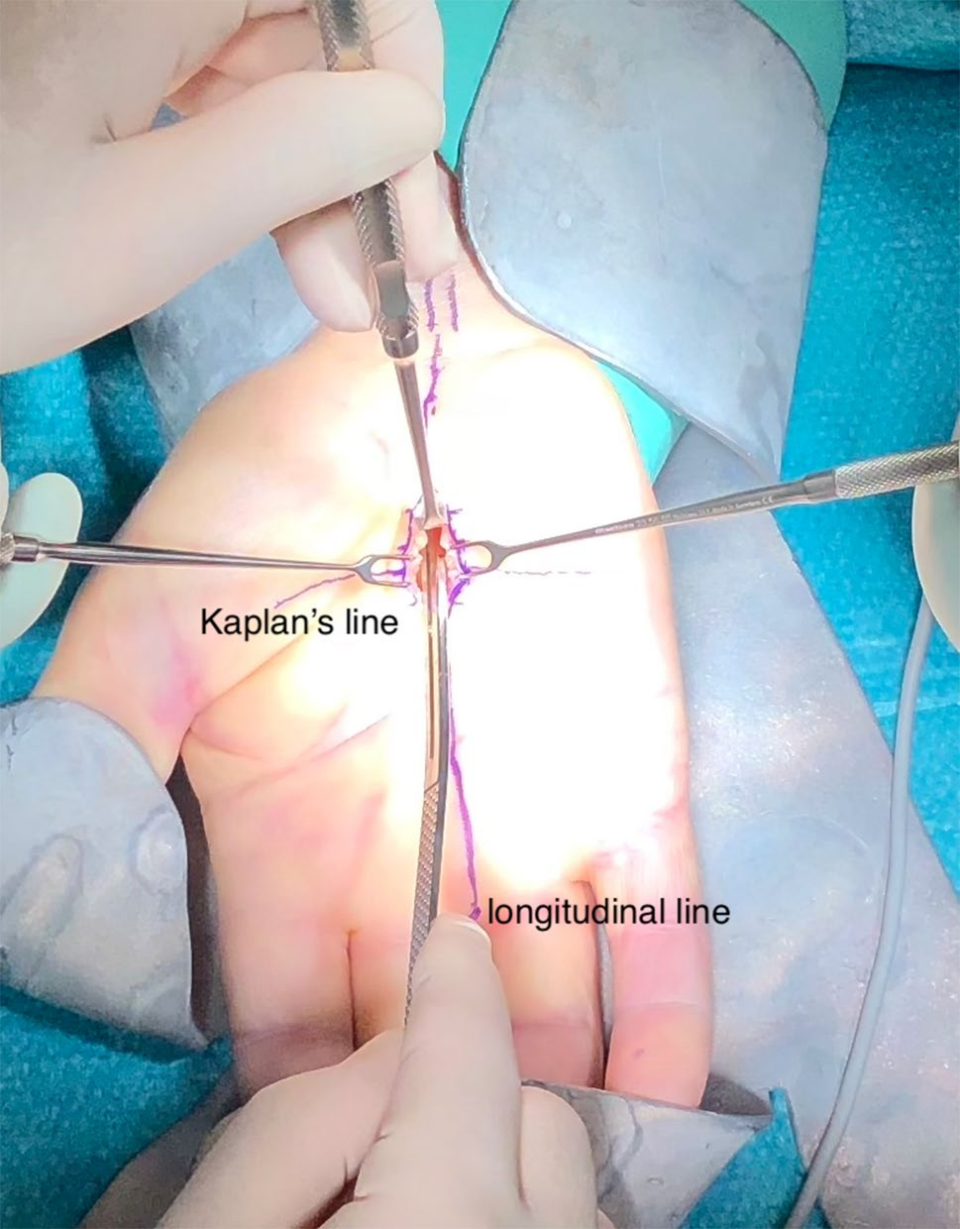
Intraoperative picture of MCTR: The protective guide has been inserted for protection of the median nerve. During exposure, two Fomon retractors are used transversely, one Ragnell retractor is used proximally. {SafeGuard Mini Carpal Tunnel Release System, Art.-No. 08-0001, INTEGRA LifeSciences Corporation, USA; MEDICON eG, Art.-No. 20.50.05, Art.-No. 20.12.20, Germany}. *MCTR* mini-open carpal tunnel release.

**Figure 6:**
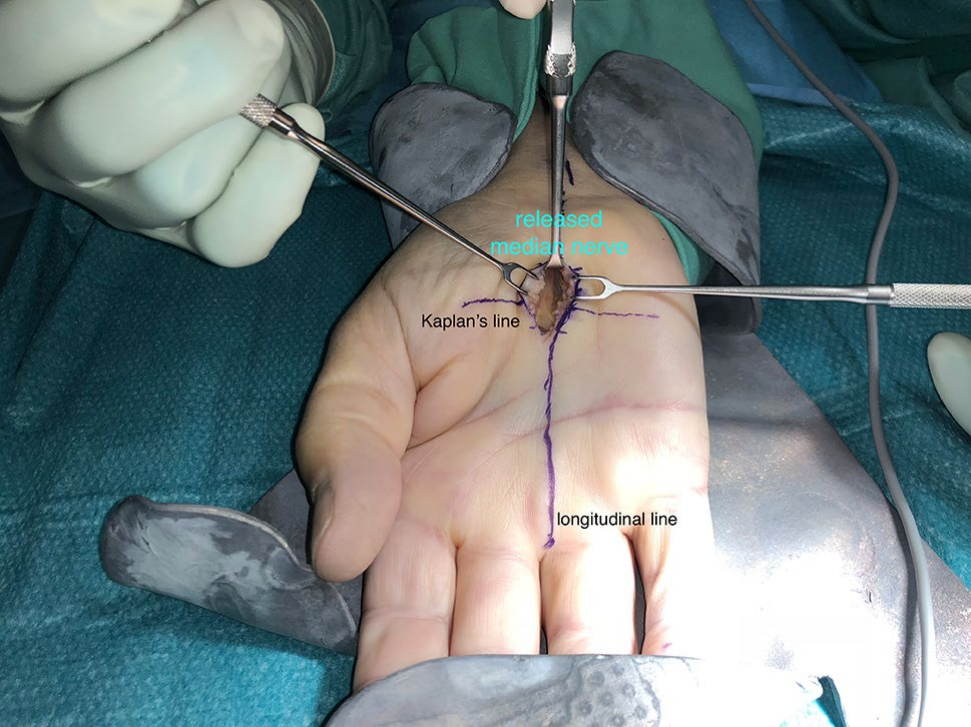
Intraoperative picture of the MCTR: Release of the transverse carpal ligament is achieved. The median nerve can be visualized ensuring its complete release. The final examination for further perineural scar tissue can be applied without any problems. {MEDICON eG, Art.-No. 20.50.05, Art.-No. 20.12.20, Germany}. *MCTR* mini-open carpal tunnel release, *OCTR* Open carpal tunnel release.

The first incision of the distal portion of the transverse carpal ligament was performed under direct vision with a surgical scalpel blade number 15 {Dahlhausen & Co. GmbH, Köln, Germany}. Next, the protective guide was inserted into the carpal tunnel in a proximal direction underneath the remaining transverse carpal ligament. The special cutting knife was inserted into its groove and passed proximally until a complete release of the remaining transverse carpal ligament has been accomplished (see Figs. 3, 5 and 6).

### Reevaluation

Retrospectively evaluated data were collected prospectively in a dedicated hospital database. Basic characteristics, which involved age, sex, affected side, duration of surgery and pre-surgical symptoms were recorded from the respective patient files.

Patients had a follow-up visit at two weeks following surgery. The early follow-up was assessed by the respective surgeon (MP or AK). Here, the presence of wound healing disturbances, infection, scar sensibility and the respective Visual Analogue Scale (VAS) were evaluated.

The final follow-up was done by two trauma surgeons (AS and GH), documenting changes in symptoms and signs and/or adverse events. During the final follow-up, all patients completed the VAS, the Disabilities of Arm, Shoulder and Hand Score (DASH) and the Boston Carpal Tunnel Syndrome Questionnaire including Symptom Severity Scale (SSS) and the Functional Status Scale (FSC). Additionally, the patients were questioned to the occurrence of scar sensibility and/or pillar pain at the site of the operation. The evaluation of scar sensibility was defined as follows: The patients were questioned about complaints at the area of the scar like burning discomfort, hypersensitivity or superficial pain during palpation. Scar sensibility was classified as the presence of one or more symptoms. Pillar pain was defined as deep-seated pain of the thenar and/or hypothenar region, related to the use of the hand like tight gripping. During examination, pillar pain was tested by simultaneous compression of thenar and hypothenar eminences as if to separate the carpal tunnel.

The durance from surgery to restitution and return to workplace (retirees excluded) was assessed. Restitution was defined as the subjective time following surgery when patients were able to perform their activities of daily living painlessly. The occurrence of adverse events (hematoma, infect, neurovascular or tendon lesions) and recurrence were evaluated. Recurrence was constituted by the return of symptoms after a temporary period of resolution and/or the need for a symptom-associated reoperation. Further, the revision rate and cause as well as the time interval from primary surgery to the revision surgery, if applicable, were assessed.

### Statistical analysis

Statistical analyses were performed using the SPSS software {IBM SPSS Statistics version 26, Armonk, USA}. Continuous parameters were presented as mean, standard deviation (SD), and categorical or quantitative data. Descriptive statistics were used for demographic variables; the continuous variables were summarized using SD and/or range via minimum and maximum.

Non-parametric tests were used for data analysis regarding significance. To investigate the differences between the MCTR and OCTR group, Mann–Whitney U tests were utilized. Chi-square tests were used to compare the remaining targets and/or adverse events. P-values (*p*) below 0.05 were set as statistically significant, confidence intervals of 95% were computed. A post-hoc power analysis was performed with G*Power 3.1.^18^. According to an alpha of 0.05, it was calculated that the sample size could achieve a power of 0.88 based on a two-tailed significance test^19^.

### Ethical details

 Ethical approval was obtained from the institutional review board of the Austrian Workers' Compensation Board (AUVA-EK 03/2019). While included, the patients consented to the study protocol and informed consent was obtained for research purposes. All the experimental protocols and methods were carried out in accordance with the regulations and principles of the Declaration of Helsinki and the ICH-GCP Guidelines.

## Results

### Basic characteristics

The MCTR group involved 72% female (36/50) and 28% (14/50) male patients with a mean age of 61.2 years (SD: 13.3; range: 36–81) at the time of surgery. Sixty-four percent (32/50) were right and 36% (18/50) left hands. The average durance of symptoms had been 4.9 months (SD: 2.2; range: 3–12). The mean durance of surgery was 9.2 min (SD: 2.7; range: 6–18). The MCTR group had a mean final follow-up of 60 months (SD: 23.1; range: 36–108).

Seventy-four percent (37/50) were female and 26% (13/50) were male patients in the OCTR group. The mean age was 59.0 years (SD: 16.7; range: 20–84) at the day of surgery. In 62% (31/50) the right and in 38% (19/50) the left hand underwent CTR. The surgical time averaged 12.0 min (SD: 3.3; range: 7–21). The average durance of symptoms had been 5.4 months (SD: 1.8; range: 4–12). The mean final follow-up was 54 months (SD: 24.3; range: 37–101) in OCTR group.

There were statistically significant differences between the durance of surgery (p = 0.001), but no statistically significant differences between the groups regarding age (p = 0.621), and time of follow-up (p = 0.623).

### First follow-up

During the follow-up visit two weeks after surgery, no adverse events were observed in both groups. Scar sensibility was present in three cases (MCTR: 3/50; 6%) and the VAS averaged at 1.4 points (SD: 2.1; range: 0–7) in the MCTR group. In the OCTR group, scar sensibility was evaluated in 13 cases (OCTR: 13/50; 26%) and the VAS was rated with 1.7 points on average (SD: 2.8; range: 0–8). Comparing the two groups in this early follow-up, the scar sensibility was statistically significant decreased following MCTR (MCTR: 3/50; 6% versus OCTR: 13/50; 26%; p = 0.002). There were no statistically significant differences regarding VAS (p = 0.327).

### Final follow-up (clinical results and adverse events)

None of the evaluated scores differed statistically significantly between the groups (see Table 1). All postoperative progresses can be overlooked in Table 2. Of these, scar sensibility was significantly decreased (p = 0.007) in the MCTR group (0%) when compared to the OCTR group (12%, 6/50).

**Table 1:** Functional differences of MCTR versus OCTR.

	DASH (in points)	SSS (in points)	FSC (in points)	VAS (in points)
MCTR	4.6 (SD: 12.9;range 0–61)	1.3 (SD: 0.9; range 1–7)	1.3 (SD: 0.7; range 1–3.8)	0.4 (SD: 1.6; range 0–7)
OCTR	8.3 (SD: 18.3; range 0–70)	1.2 (SD: 0.6; range 1–6)	1.2 (SD: 0.6; range 1–5.7)	0.7 (SD: 1.9; range 0–7)
	p = 0.398	p = 0.534	p = 0.617	p = 0.246

Functional results are listed at mean. All scores were statistically not significant in comparison.

*DASH* disabilities of arm, shoulder and hand score, *SSS* symptom severity scale, *FSC* functional status scale, *VAS* visual analogue scale, *MCTR* mini-open carpal tunnel release, *OCTR* Open carpal tunnel release.

**Table 2:** Postoperative follow-up of MCTR versus OCTR.

	Scar sensibility (in cases)	Pillar pain (in cases)	Restitution (in weeks)	Return to work (in days)
MCTR	0	0	10 (SD: 13.2; range 0–52)	14 (SD: 12.3; range 2–49)
OCTR	12% (6/50)	6% (3/50)	15 (SD: 19.6; range 0–78)	20 (SD: 15.2; range 4–56)
	p = 0.007	p = 0.268	p = 0.185	p = 0.142

Specific follow-up comparisons show a statistically significant superiority of the MCTR group in terms of scar sensibility. Restitution and return to work are presented at mean.

*MCTR* mini-open carpal tunnel release, *OCTR* Open carpal tunnel release.

The perioperative adverse events are listed in Table 3. In both groups, no recurrences were to observe. The revision rate was 2% in the MCTR group (1/50) and 4% in the OCTR group (2/50). In both samples, patients suffering from an infection underwent one singular revision surgery. The mean time from primary to revision surgery was two weeks for the MCTR and 6.3 weeks (range: 3.5–9) for the OCTR group.

**Table 3:** Perioperative adverse events per group (in cases—multiple events are possible per patient).

	Hematoma	Infection	Nerve lesion	Vessel lesion	Tendon lesion
MCTR	0	2% (1/50)	2% (1/50)	0	0
OCTR	0	4% (2/50)	0	0	0
	p = 1.0	p = 0.851	p = 0.674	p = 1.0	p = 1.0

Complications are listed regarding MCTR versus OCTR. There were no statistically significant differences in the frequency of adverse events observed in either group.

*MCTR* mini-open carpal tunnel release, *OCTR* Open carpal tunnel release.

In the overall collective, no iatrogenic vascular, nerve branch or tendon injuries were documented. One partial median nerve lesion on the palmar aspect was to verify in the MCTR group, following by extending the incision and direct nerve repair via micro-neurosurgical technique. A full nerve function could be reached two years postoperatively, which was evaluated clinically and via electromyogram testifying. In total, the overall complication rate amounted to 4% (4/100 in total or 2/50 per group).

## Discussion

The aim of this study was to evaluate the long-term follow-up of a minimally-invasive CTR through a palmar approach and to compare its outcomes to the conventional procedure. In our sample, none of the functional scores differed significantly between the groups. The scar sensibility was statistically significantly decreased following MCTR when compared to OCTR in the early- (p = 0.002) and long-term follow-up (p = 0.007).

Paine^20^ has descript the first device used as a retinaculotome in 1955. This device has been used up to now, as Fernandes et al.^21^ reported more recently. The authors^21^ observe short- and long-term results and reported good clinical results of more than 500 patients in a period of 17 years. The palmar incision for CTR has been used for a long time, various strategies have been reported over the time. Aryan et al.^22^ reported 1977 a representative case series of 429 patients with improved symptoms. Using a similar retinaculotome, our findings address a lack in the literature. Existing studies^23,24^ are scarce regarding long-term outcomes following MCTR.

Bai et al.^2^ performed a retrospective analysis of prospectively collected data concerning 85 patients who had either undergone MCTR or OCTR including similar incisions as performed in the current study. The average duration of symptoms had been 6.6 months (MCTR) and 6.4 months (OCTR), respectively, which is comparable to our sample (MCTR: 4.9 months; OCTR: 5.4 months). Mean durance of surgery did not differ significantly (p = 0.130) between the groups (MCTR: 25.1 min; OCTR: 23.5 min), which was different in our collective (MCTR: 9.2 min; OCTR: 12.0 min; p = 0.001). As in our sample, the authors found no statistically significant differences regarding VAS and DASH between the groups (VAS: p = 0.246, DASH: p = 0.398). At twelve months follow-up, the rate of scar pain was at 4.7% in the OCTR group, whereas none of the patients suffered from wound pain following MCTR (p = 0.490). Similarly, scar sensibility was significantly increased (p = 0.007) in the OCTR group (12%) when compared to our MCTR group (0%) in our long-term outcome.

Aslani et al.^25^, divided their sample of 105 patients into three subgroups (MCTR, OCTR and a group undergoing endoscopic CTR). The average interval of return to work was significantly (p =  < 0.05) longer in the OCTR (mean: 21.1 days) when compared to the MCTR (mean: 12.7 days). We also evaluated a shorter durance of absence at workplace and a non-statistical significant superiority of the MCTR group (MCTR: mean 14 days; OCTR: mean 20 days; p = 0.142). This could be considered as positively cost-effective on the basis of an earlier return to work.

Zhang et al.^23^ performed an analysis of 207 patients who were randomized into a MCTR group through two small incisions (n = 73), an OCTR group (n = 65) and a group that underwent endoscopic CTR (n = 69). The mean duration of symptoms had been six months in the MCTR and OCTR group, which is well comparable to our sample (MCTR: 4.9 months; OCTR: 5.4 months). At the final follow-up of three years, there were no statistically significantly differences between MCTR and OCTR regarding outcomes of the Boston Carpal Tunnel Syndrome Questionnaire. Here, the SSS and FSC were at a mean of 1.2 points in both groups. These values are well comparable to our results.

Recurred nerve compression occurs at a rate of less than 2% to as high as 25%^26–31^ and may happen years after surgery^30^. Recurrence rates in long-term follow-up studies are reported from 3.7%^27^ to 57%^32^ of cases. However, there is a lack of consistent definition for recurrence in the literature, which may explain this range. Some authors define the return of any preoperative symptoms^32^, others the need for reoperation^27^ to qualify as recurrence. Cresswell et al.^24^ reported a higher rate of immediate complications, and more recurrences in patients following MCTR compared to the conventional procedure. The authors^24^ assessed results seven years postoperatively. In contrast, no recurrences were to observe in both of our groups with a minimum follow-up of three years and a shorter final follow-up (mean final follow-up of MCTR: 60 months and OCTR: 54 months). This might be traced back to our shorter follow-up intervals and the bias on the returned questionnaires of Cresswell et al.^24^, which may not reflect the entire cohort.

Common and main indications of recurrences of nerve compression may cause the incomplete splitting of the transverse ligament and/or a postoperative fibrosis^30,33^. It is to believe, that the postoperative fibrosis may positively influenced, like with our early motion protocol from the first postoperative day on. Complete division of the carpal ligament is a factor associated with surgery. Kilinc^34^ showed, that recurrent CTS after sufficient division of the transverse ligament is very unlikely. Additionally, similar low adverse events were observed in both groups. Anyhow, these facts indicate the positive effectiveness and permit the inference, that MCTR represents a reliable method for CTR.

One further technical advantage represents the direct visualization of the thenar branch of the median nerve. Further, no iatrogenic lesions of the superficial palmar arterial arch can occur due to the proximally directed release. In this regard, we highlight, that we had to observe one partial median nerve laceration in the MCTR group in one patient with high-grade adhesions around the median nerve. Similarly, Cresswell et al.^24^ reported one lesion of the median nerve in 53 patients. Lee and Strickland^17^ observed two median nerve lesions in their 694 releases with the retinaculotome. Summing up our findings and both studies, the median nerve lesion is a noteworthy major complication, whereby the rate represents less than 1% in all three research groups. Based on our observations and experiences, we strictly recommend to enlarging the skin incision in patients with high-grade adhesion situations.

This study had several limitations. We solely compared MCTR with OCTR and did not include an endoscopic group. Moreover, no randomized assignment was calculated for the respective groups. Furthermore, although we used prospectively collected data, the study remains retrospective nature. Randomized controlled trials would be recommendable to re-evaluate this technique in future. Minimally-invasive procedures are generally preferred by patients, although patients as well as hand surgeons may have decisional conflicts about the treatment^35^. These factors were not addressed in the study. Lastly, financial implications were not reviewed regarding material costs or the localization of doing the surgery or inpatient versus outpatient setting.

As strength of this study is the long-term timeframe to entitle. To substantiate the operative technique in special, we used a matched-patient as well as single-surgeon study design with experienced practitioners. Moreover, the surgical procedure is easily reproducible, because it is based on superficial anatomical landmarks. The further magnitude of this minimally-invasive method represents the simple approach and the intraoperative visibility of both structures at danger—namely the thenar branch of the median nerve and the superficial palmar arterial arch. Our method was developed under inclusion of well-known anatomical safe zones^15–17^ and anatomical landmarks for a mini-open technique as proven by Hohenberger et al. in an anatomical study^14^.

In conclusion, our suggested and preferred MCTR technique through a palmar approach has been demonstrated to be effective. Patients following MCTR reached the same functional long-term outcome as the conventional procedure. The MCTR procedure has a low complication rate, both techniques are comparable in this respect. It is to recommend to enlarging the skin incision in patients with high-grade adhesions.

With the technique we described, no recurrences or patients suffering from pillar pain were to observe. Additionally, it combines the advantages of a reduced recovery period and surgical time. The major patient-specific benefit represented the decreased scar sensibility due the small 1–1.5 cm incision and its localization, which convinced as statistically significant when compared to the OCTR group. These positive effects might be traced back to the reduced damage of soft tissues while preserving the adjacent structures attributed. Thus, MCTR is a fast, practicable, minimally-invasive and minor technically challenging procedure, which allows a direct visualization of anatomical structures at risk.
